# EDUCATION AS A MEDIATOR THROUGH THE EFFECT OF AGE AT MIGRATION ON COGNITIVE HEALTH AMONG OLDER IMMIGRANTS IN THE US

**DOI:** 10.1093/geroni/igae098.2872

**Published:** 2024-12-31

**Authors:** Dongfang Hong, Wuyi Dong

**Affiliations:** University of Massachusetts Boston, Boston, Massachusetts, United States; University of Massachusetts Boston, Boston, Massachusetts, United States

## Abstract

**Background:**

Age at immigration is a critical determinant that influences long-term cognitive outcomes among older adults, often through disruptions or enhancements in educational opportunities. Previous research has underscored the importance of educational attainment as a key factor in cognitive resilience and decline. The mediating role of education in the relationship between the timing of immigration and cognitive function remains underexplored. This study addressed whether age at migration is associated with cognition among older immigrants whether education mediates this association?

**Methods:**

Data were taken from the 2020 Health and Retirement Study (N=1,587). Linear regression is employed to examine the direct association between age at migration and cognitive function and the indirect association through years of completed education.

**Results:**

This study revealed a significant association between age at immigration and cognitive function, with immigrants who migrated during young adulthood (ages 18-40) displaying notably lower cognitive function compared to those who migrated at younger ages (before 18). The results also provided evidence for the partial mediation of educational attainment for the association between age at immigration and cognitive function. This indicated that disruptions in education due to migration contribute to cognitive outcomes.

**Discussion:**

This study contributed to our understanding of how immigration timing and educational attainment intersect to affect cognitive function, with implications for policy and intervention strategies aimed at supporting the cognitive health and educational opportunities of immigrant populations. Future research should consider additional mediators, such as socioeconomic status and social support, to further elucidate these relationships

